# Viral Replication and Progression of Cancer

**DOI:** 10.2174/1874357901812010014

**Published:** 2018-02-28

**Authors:** Guest Editor (s), Arnolfo Petruzziello

**Affiliations:** Virology and Molecular Biology Unit, Istituto Nazionale Tumori, IRCCS Italia, Fondazione G.Pascale, 80131, Via Mariano Semmola, Naples (Italy), Tel: +39 0815903433/ 373/ 1779 a.petruzziello@istitutotumori.na.it

**Keywords:** Hepatitis C virus, HCV Genotypes, Hepatitis B virus, Hepatocellular carcinoma, Chemotherapy, Viral reactivation, Cirrhosis

## AIM & SCOPE

It has been widely established that several virus infections are strictly correlated to the cancerogenesis and may induce, through their reactivation, severe damages during chemotherapy treatments. For example, the direct role that Hepatitis B virus (HBV) plays in the development of liver cancer is well known. Nevertheless, in other cases, as Hepatitis C virus (HCV) or Hepatitis Delta Virus (HDV) the direct role of viral replication in cancer progression is still controversial, especially in some areas, like Italy, where these infections are endemic.

Aim of this project is to up date the epidemiological status of the incidence of HCC in Europe and HCV and HDV infection , with special attention to the changes during the time of route of transmission, and also investigate the interaction between viral reactivations and tumors.

## 1 HCV AND HEPATOCELLULAR CARCINOMA: PATHOGENETIC MECHANISMS AND IMPACT OF DAA’S

Ivan Schietroma^1^, Giuseppe Corano Scheri^1^, Claudia Pinacchio^1^, Maura Statzu^2^, Arnolfo Petruzziello^3^

^1^Department of Public Health and Infectious Diseases, "Sapienza" University of Rome, Rome, Italy;

^2^Department of Molecular Medicine, Laboratory of Virology, "Sapienza" University of Rome, Italy;

^3^Virology and Molecular Biology Unit , Department of Pathology, Istituto Nazionale Tumori- IRCCS Fondazione G. Pascale, Naples (Italy)

Corresponding author: Giuseppe Corano Scheri

(giuseppe.coranoscheri@uniroma1.it ; +393896943773)

### Reviewer

# 1 Stefano Rusconi - Clinic of Infectious Diseases, DIBIC Luigi Sacco, Milan

*# 2 Massimo Ciccozzi* - *Department of Infectious, Parasitic and Immunomediated Disease, National Institute of Health, Rome*

## 2 EPIDEMIOLOGY OF HEPATITIS B VIRUS (HBV) AND HEPATITIS C VIRUS (HCV) RELATED HEPATOCELLULAR CARCINOMA

Corresponding author: Arnolfo Petruzziello

Virology and Molecular Biology Unit, Department of Pathology, Istituto Nazionale Tumori- IRCCS Fondazione G. Pascale, Naples (Italy)

EMAIL: a.petruzziello@istitutotumori.na.it

### Reviewer

*# 1 Nicola Coppola* - *Section of Infectious Diseases, Department of Mental health and Public Medicine, University of Naples “Luigi Vanvitelli”, Italy.*

# 2 Carolina Scagnolari- Virology – Department of Molecular Medicine- University La Sapienza, Rome , Italy

## 3 DETERMINING THE ACTUAL PREVALENCE OF HEPATITIS B IN KHYBER PAKHTUNKHWA-PAKISTAN: A META-ANALYSIS

Najeeb Ullah Khan^1^, Ali Zalan^1^, Arnolfo Petruzziello^3^, Iftikhar ud din^2^, Fazle Haq^2^, Yousaf Hayat^2^

^1^ Institute of Biotechnology and Genetic Engineering (Health Division), The University of Agriculture Peshawar, Pakistan

^2^ Department of Mathematics, Stats & Computer Science, The University of Agriculture Peshawar, Pakistan

^3^ Virology and Molecular Biology Unit “V. Tridente”, Department of Pathology, Istituto Nazionale Tumori- IRCCS Fondazione G. Pascale, Naples (Italy) 

Corresponding author: Najeeb Ullah Khan^1^

E-mails: najeebkhan@aup.edu.pk, naji_banni@yahoo.com

### Reviewer

# 1 Maria Lina Tornesello, PhD- Molecular Biology and Viral Oncology, IRCCS, Fondazione Pascale, Naples, Italy

# 2 Sabrina Bimonte, Md- Division of Abdominal Surgical Oncology, Hepatobiliary Unit, National Cancer Institute , IRCCS Italia G.Pascale, Naples (Italy)

